# CD47 regulates antigen modulation and red blood cell clearance following an incompatible transfusion

**DOI:** 10.3389/fimmu.2025.1548548

**Published:** 2025-04-04

**Authors:** Ryan P. Jajosky, Mischa L. Covington, Jun Liu, Li Chai, Patricia E. Zerra, Satheesh Chonat, Sean R. Stowell, Connie M. Arthur

**Affiliations:** ^1^ Joint Program in Transfusion Medicine, Department of Pathology, Brigham and Women’s Hospital, Harvard Medical School, Boston, MA, United States; ^2^ Harvard Glycomics Center, Harvard Medical School, Boston, MA, United States; ^3^ Aflac Cancer and Blood Disorders Center, Children's Healthcare of Atlanta, Atlanta, GA, United States; ^4^ Department of Pediatrics, Emory University School of Medicine, Atlanta, GA, United States; ^5^ Center for Transfusion and Cellular Therapies, Department of Pathology and Laboratory Medicine, Emory University School of Medicine, Atlanta, GA, United States

**Keywords:** red blood cell, alloimmunization, incompatible transfusion, antibodies, antigen modulation

## Abstract

Red blood cell (RBC) alloantibodies can result in the rapid removal of incompatible RBCs following transfusion. However, antibody-mediated clearance of RBCs is not the inevitable outcome of an incompatible transfusion. Antibody engagement can also result in the modulation of the target antigen, often rendering RBCs resistant to antibody-mediated removal. Despite this, the factors that regulate antibody-induced RBC removal or antigen modulation remain incompletely understood. Given the ability of CD47 to regulate RBC survival in general, we examined the possible role of CD47 in governing antibody-mediated RBC clearance and antigen modulation. This was achieved by crossing the well-established HEL-OVA-Duffy (HOD) mouse model with CD47 knockout (KO) mice to generate offspring that express the HOD antigen and either WT (HOD CD47 WT), heterozygote (HOD CD47 HET) or KO (HOD CD47 KO) levels of CD47. Using the commonly employed anti-HEL immunization model, our results demonstrate that while antibody engagement of HOD CD47 WT RBCs resulted in rapid antigen modulation in the absence of detectable RBC clearance, antibody binding to HOD CD47 HET RBCs did result in detectable RBC removal despite similar rates and overall levels of antigen modulation. In contrast, despite accelerated clearance of HOD CD47 KO RBCs in the absence of anti-HEL antibodies, the rate of RBC removal and antigen modulation was enhanced in the presence of anti-HEL antibodies. Taken together, these results suggest a role for CD47 in regulating the overall consequence of an incompatible RBC transfusion.

## Introduction

Blood transfusion represents one of the most common interventions in medicine. While red blood cell (RBC) transfusion in particular can be lifesaving, it is not without risk (1–3). Variations in RBC surface antigens between individuals can trigger the production of alloantibodies (4). While blood group antigens probably developed as a response to evolutionary pressures from infectious agents and likely play specific roles in the host’s immune system (5–11), the formation of alloantibodies against RBC alloantigens can create challenges in identifying suitable RBC units for subsequent transfusions and increase the risk of hemolytic transfusion reactions. These antibodies can also evanesce to undetectable levels when using standard blood bank testing methods. In patients who require repeated blood transfusions, such as those with certain medical conditions like sickle cell disease (SCD), this poses a significant risk (12, 13). As alloantibodies can go undetected at the time of transfusion, when patients are re-exposed to RBC alloantigens to which they have previously mounted an immune response, it can trigger a recrudescent alloimmune response that results in accelerated clearance of the transfused RBCs that constitutes a delayed hemolytic transfusion reaction (14, 15).

Perhaps in part due to distinct underlying features of immunity (16–18), in patients with SCD, this process can be even more severe (15, 19–22). Anamnestic immune responses that result from alloantigen reexposure not only target the transfused RBCs but can also result in the accelerated clearance of the patient’s own RBCs (15, 19–21). This phenomenon, known as hyperhemolysis, can lead to severe anemia and life-threatening complications, including severe organ damage and death (20, 23–29). Unlike immune-mediated platelet clearance (30, 31), antibody-induced hemolysis can cause significant complications, likely in part due to the impact of free heme on innate immunity, including complement activation (20, 24–29). These transfusion complications can limit the availability of potential long-term treatment options, such as gene therapy or bone marrow transplantation due to challenges in providing safe peri-transplant transfusion support (20, 32–34). Importantly, the impact of RBC alloimmunization on morbidity and mortality is not limited to patients with SCD, but also extends to other transfusion-dependent patient populations (35).

Multiple approaches have been adopted to minimize exposure to alloantigens in patients who require chronic RBC transfusion therapy, including antigen-matching (36), with the goal of decreasing the risk of alloimmunization and its associated complications. Early studies demonstrated that this approach could lower the incidence of immune responses against common alloantigen targets (36). However, the complexity of some alloantigen systems has presented challenges in achieving comprehensive matching for various antigenic structures (37). Recent findings support this, revealing that even with efforts to match for prevalent alloantigens and minimize exposure to alloantigens in general, patients can still develop alloantibodies (38). Consequently, there have been significant efforts aimed at discovering complementary approaches that could proactively inhibit the formation of RBC alloantibodies following alloantigen exposure (39–48). These investigations, encompassing both preclinical and clinical approaches, have shed light on the critical immune mechanisms that trigger the development of alloantibodies following RBC transfusion that may aid in preventing alloimmune responses against other intravascularly delivered antigens (49–55). Further studies have not only pinpointed significant regulators that govern this process but have also enhanced our understanding of patient-specific characteristics that affect the likelihood of antibody production after RBC transfusion (14, 38, 56–66). By identifying pathways that govern RBC alloimmunization, potential therapeutic targets have emerged that might help prevent or at least reduce the risk of alloantibody formation following RBC transfusion.

While the results of preclinical and clinical studies provide important insight into potential strategies that may be employed to actively prevent alloantibody formation, in some patient populations, 30-40% of patients have already developed alloantibodies (1, 67–69). Consequently, while there are efforts to minimize further alloantibody development, strategies focused solely on prevention may be less effective at reducing or eliminating the consequences of RBC alloimmunization in these patients. This is especially apparent when considering patients can form alloantibodies against a highly prevalent antigen or develop a complex mixture of alloantibodies that makes it difficult to find compatible RBC units (70–72). When this occurs, units that become available can remain incompatible for a given alloantigen. This situation becomes especially challenging when patients with complicated alloantibodies present with life-threatening anemia requiring urgent RBC transfusions (70–73). As current transfusion strategies are nearly uniformly aimed at providing compatible RBCs, when situations arise in which compatible units are unavailable, there are few strategies that have been developed to prevent the negative consequences that can result from an incompatible RBC transfusion.

To better define the factors that regulate incompatible transfusion, several preclinical models, utilizing distinct target antigens, have been leveraged (74–89). One of these is the HOD (HEL,OVA,Duffy) system, which incorporates the HEL and OVA model antigens by coupling them to the Duffy blood group antigen as a chimeric fusion protein (88, 89). Studies using this model have demonstrated that antibody-mediated elimination of HOD RBCs appears to occur through interaction of Fcy receptors (FcγR) (82, 90). Consistent with this, previous studies have demonstrated that anti-HOD antibodies targeting different portions of the HOD antigen fail to fix detectable levels of complement (86). However, in addition to this FcγR-dependent pathway, FcγR-independent mechanisms, have also been described (90). Interestingly, the mechanism of clearance can vary depending on the combination of antibodies that the transfused HOD RBCs encounter (77, 78, 87).

Despite many studies examining the mechanisms whereby antibodies induce RBC clearance, several studies indicate that RBCs may not always be removed following an incompatible transfusion (77, 78, 82, 90–94). While there are several possible reasons for this to occur, the ability of antibodies to induce alterations to the target antigen has emerged as a unique outcome of incompatible RBC transfusion that can occur in the absence of any detectable RBC clearance (77, 78, 82, 90–94). This phenomenon, often referred to as antibody-induced antigen modulation, can reduce the levels of detectable antibody present on the RBC surface, often rendering RBCs less susceptible to further antibody-driven clearance (77, 78, 82, 90–94). Interestingly, this antibody-dependent decrease of antigen can also occur in either an FcγR-dependent or -independent manner (77, 78, 87). However, the factors that govern antibody-mediated loss of antigen remain relatively unknown.

Among regulatory proteins on the cell surface, RBCs express CD47, an anti-phagocytic marker expressed on a wide range of cell types (95–97). CD47 is known to prevent cellular removal via its interaction with SIRPα^98-100^. This interaction has been extensively studied, particularly in the context of cancer cells, which can upregulate CD47 to block immune-mediated phagocytic removal (98). Given the critical role of CD47 in the regulation of cellular removal, levels of CD47 on transfused RBCs may play a critical role in the outcome of incompatible transfusion. Indeed, an interplay between antibody-induced RBC removal and CD47-mediated signals may in part dictate whether antibody-induced antigen modulation or RBC removal occurs following antibody engagement of transfused RBCs.

Given the consequences of incompatible RBC transfusion on patient outcomes, we sought to investigate the role of CD47 as a regulator of antibody-mediated antigen modulation and RBC clearance. To accomplish this, we bred HOD RBC mice with CD47 knockout (KO) mice to create three types of donor RBC populations: one with normal CD47 levels (HOD CD47 WT), one with half the normal levels (CD47 heterozygous; HOD CD47 HET), and one completely lacking CD47 (HOD CD47 KO). Despite possessing similar HOD levels, these three donor RBCs showed varying levels of susceptibility to antibody-mediated RBC clearance. While HOD CD47 WT and HOD CD47 HET RBCs did not exhibit significant clearance following transfusion into non-immunized recipients, HOD CD47 HET RBCs were uniquely sensitive to anti-HEL-mediated RBC removal. In contrast, HOD CD47 KO RBCs exhibited accelerated clearance following transfusion into non-immunized recipients, which was further enhanced and accompanied by accelerated antigen loss following transfusion into anti-HEL immunized recipients. These findings reveal a role for CD47 in controlling both RBC clearance and antigen loss in response to incompatible transfusion, offering important insights into the mechanisms affecting RBC fate following antibody engagement.

## Methods

### Mice

C57BL/6 (B6) mice were purchased from Charles River Laboratories. B6.129P2-Fcer1gtm1Rav N12 (FcRy constitutive knockout, model 583) mice were obtained from Taconic. HOD transgenic mice were generated as described previously (99). These mice express the triple fusion protein HOD (hen egg lysozyme, a portion of ovalbumin, and Duffy b antigen) on RBCs (via a beta-globin promoter). CD47 deficient mice (B6.129S7-Cd47tm1Fpl/J) were purchased from The Jackson Laboratory. HOD mice were then crossbred with CD47KO mice to create: HOD CD47WT, CD47HET, and CD47KO mice. The Division of Animal Resources and Husbandry at Brigham and Women’s Hospital bred and housed these mice. Experiments were initiated in male and female (approximately 1:1 ratio) mice ages 8-12 weeks old. Each experiment included 3-5 mice per group, with representative data of 3 experiments shown. The experimental protocols and animal procedures described herein were approved by the Institutional Animal Care and Use Committee (IACUC).

### Antibodies

APC-conjugated anti-CD47 antibody (clone miap301), anti-CD71 (clone RI7217) and APC-conjugated anti-Ter119 (Ly-76) were obtained from Biolegend. The anti-HEL monoclonal antibodies (2F4 and 4B7), which are mouse IgG1, were obtained from BioXcell. APC-conjugated goat anti-mouse IgG was obtained from Jackson ImmunoResearch Laboratories. Biotinylated anti-mouse C3 (clone RmC11H9) and biotinylated anti-mouse iC3b (clone 10C7) were obtained from Cedarlane. APC-conjugated streptavidin was obtained from Biolegend.

### RBC transfusions and antigen level evaluation

HOD and B6 RBCs were labeled with DiI and DiO, respectively, and transfused as previously described (77, 78, 82). Briefly, HOD antigen positive and negative RBCs are labeled with unique fluorescent dyes and mixed at a 1:1 ratio before transfusion into designated recipients as outlined previously (77). Following transfusion, blood samples are assessed at key timepoints and the ratio of antigen positive to negative RBCs is calculated. Multiple time-points are selected to assess the progression of antigen specific RBC clearance, and the 4h time-point is shown to highlight the earliest time-point at which significant differences in clearance are observed. In all clearance experiments, percent survival is calculated by determining the ratio of HOD antigen positive cells to antigen negative control cells transfused together. For normalization of RBC clearance, percent clearance for each group is normalized to its relevant PBS control group. RBC stains were performed as previously described (83, 100). In brief, RBCs were washed three times with phosphate buffered saline (PBS) prior to staining. Then, a saturating concentration of the primary antibody was added and incubated for 15 minutes at room temperature, followed by three washes with PBS. Then, a saturating concentration of the secondary antibody was added if needed, followed by washing three times with PBS. Reticulocytes were distinguished from mature RBCs in whole blood from each donor by CD71 expression, as described previously (51). A Cytek NorthernLights flow cytometer was used to acquire the data followed by data analysis using FlowJo software.

### Statistical analyses

GraphPad Prism software was used for statistical analysis. Two groups were analyzed using a student’s t tests, while ≥3 groups were analyzed using a one-way analysis of variance (ANOVA) with Tukey multiple comparison test. P ≤.05 was considered statistically significant.

## Results

**Figure 1:**
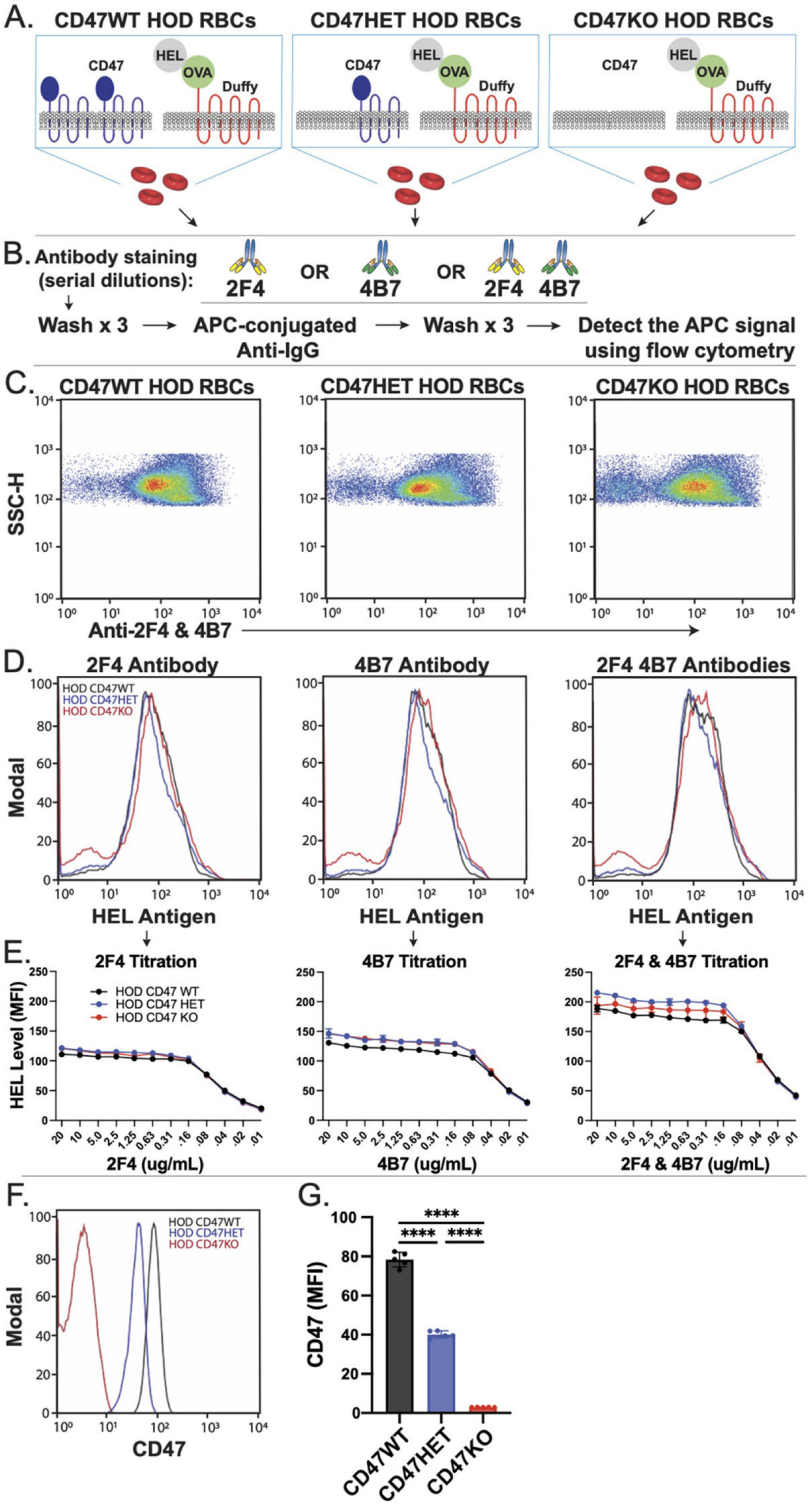
CD47 knockout and heterozygous HOD RBCs possess reduced levels of CD47 with comparable HOD antigen levels. **(A)** Depictions of distinct HOD CD47 RBC populations used in this model. **(B)** Protocol for detecting HEL antigen on the surface of HOD CD47 RBCs. **(C)** Examination of HEL antigen expression on HOD CD47 WT, HOD CD47 heterozygous (HET) or HOD CD47 knockout (KO) shown by dot plots examining anti-HEL antibody (2F4 or 4B7) engagement by side scatter (SSC). **(D)** Evaluation of anti-HEL antibody binding over a range of concentrations toward HOD CD47 WT, HOD CD47 HET and HOD CD47 KO RBCs by histogram. **(E)** Quantification of histogram mean fluorescent intensity (MFI) values using a range of concentrations of anti-HEL antibodies individually or in combination toward HOD CD47 RBCs WT, HOD CD47 HET and HOD CD47 KO RBCs. **(F)** Histogram analysis of CD47 expression on HOD CD47 WT, HOD CD47 HET and HOD CD47 KO RBCs. **(G)** Quantification of histogram MFI values of CD47 antigen expression on HOD CD47 WT, HOD CD47 HET and HOD CD47 KO RBCs. ****p < 0.0001.

To test the hypothesis that a balance between RBC removal and antigen loss post-antibody engagement may in part be influenced by CD47, we bred CD47 KO mice, which lack CD47 entirely, with HOD mice across several generations to create donor mice with varying CD47 levels (wild type, heterozygote, or knockout) (**Figures 1A, B**). We first assessed HOD antigen levels in each donor group (HOD CD47 WT, HOD CD47 HET, and HOD CD47 KO), using a set of monoclonal antibodies that target distinct epitopes on the HEL antigen. Using this approach, no significant differences in HEL levels among the donor groups was observed. This was consistent regardless of whether one or a combination of these monoclonal antibodies was used, suggesting that HEL antigen levels remain comparable across CD47 WT, CD47 het, and CD47 KO HOD RBC donors (**Figures 1B-E**). We also examined the composition of the peripheral blood collected from each RBC donor. Interestingly, blood collected from CD47 HOD KO donors did contain a slightly higher percentage of reticulocytes than blood collected from CD47 HOD WT donors, and reticulocytes in general exhibited higher levels of HOD antigen expression across all donors. However, no differences in the level of HOD antigen were observed between donors when comparing levels on reticulocytes or mature RBCs (**Supplementary Figure 1**). This difference in reticulocyte count was insufficient to cause differences in overall HEL levels on RBCs collected from each of the three donors (**Supplementary Figure 1**; **Figures 1B-E**), possibly due to the subtle differences in reticulocytes numbers between each group where CD47 HOD KO may have 1% more reticulocytes among the total RBC population than some CD47 HOD WT donors.

We next sought to ensure that the apparent lack of differences in the HEL antigen on the surface of HOD CD47 WT, CD47 HET and CD47 KO was not due to incomplete saturation of the anti-HEL antibodies used to define HEL levels on the RBC surface. To accomplish this, we titrated each monoclonal antibody over a range of concentrations against each of these three HOD RBC types. Using this approach, comparable levels of HEL on the CD47 WT, CD47 het, and CD47 KO HOD RBCs were observed across a wide range of antibody concentrations (**Figure 1E**). Additional examination of the HOD antigen using a combination of anti-HEL antibodies yielded similar results. To verify if the offspring of each RBC donor had the expected CD47 levels, we analyzed CD47 on RBCs from each group. As seen in prior studies (95), CD47 expression varied based on gene dosage (**Figures 1F, G**). RBCs from mice heterozygous for CD47 (HOD CD47 HET) expressed roughly half the CD47 expression levels observed from homozygous donors (HOD CD47 WT) donors. Conversely, RBCs from CD47 KO mice had no detectable CD47 (**Figures 1F, G**). These findings indicate that crossing the HOD RBC model system with CD47 KO mice can produce distinct donor RBCs with WT, HET or KO levels of CD47 while retaining similar levels of the HOD antigen.

**Figure 2:**
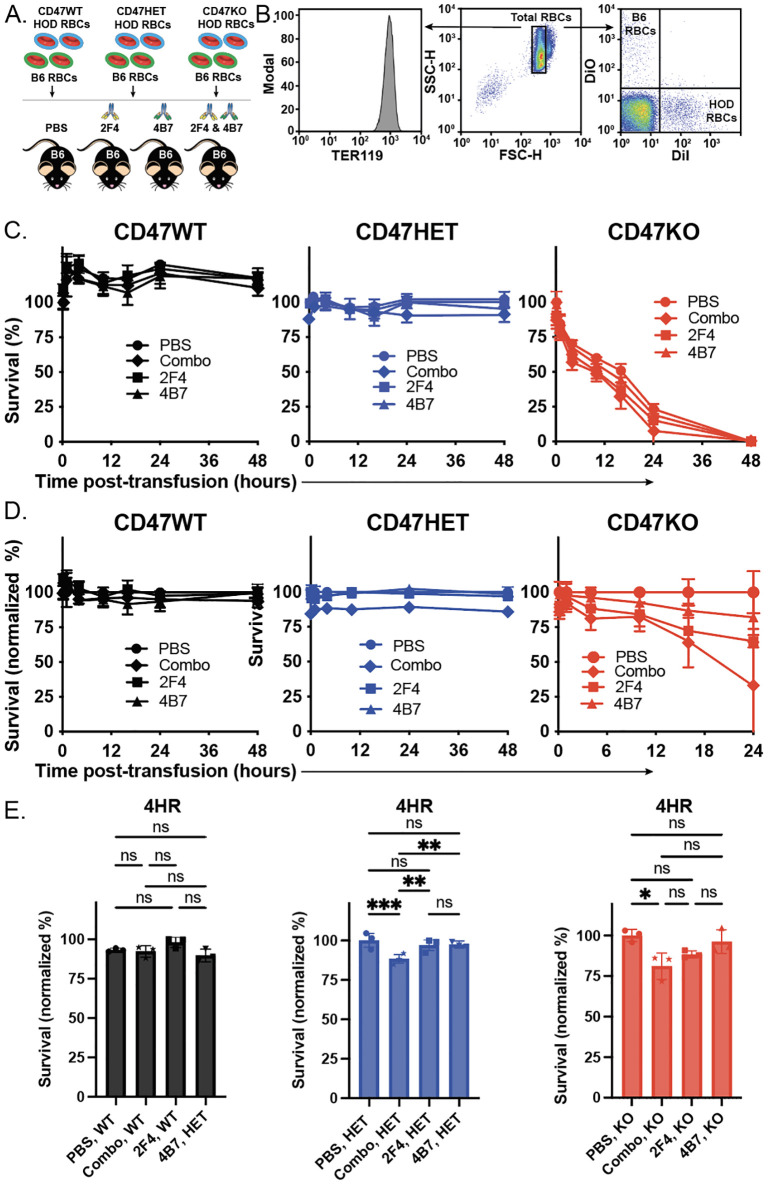
Anti-HEL antibodies enhance clearance of HOD CD47 heterozygous and knockout RBCs, while failing to impact HOD CD47 WT RBC removal. **(A)** Depiction of the experimental design using HOD CD47 WT, HOD CD47 heterozygous (HET) or HOD CD47 knockout (KO) RBCs transfused into non-immunized (PBS) or recipients immunized with one (2F4 or 4B7) or a combination (2F4 and 4B7) of anti-HEL antibodies. **(B)** Flow cytometry gating strategy used to detect DiI labeled HOD CD47 WT, HOD CD47 HET and HOD CD47 KO RBCs or DiO labeled B6 RBCs following transfusion into non-immunized or anti-HEL immunized recipients. **(C)** Survival of transfused HOD CD47 WT, HOD CD47 HET and HOD CD47 KO RBCs over time in comparison to B6 RBCs in the same recipient. **(D)** Normalized survival of transfused HOD CD47 WT, HOD CD47 HET and HOD CD47 KO RBCs over time in immunized recipients in comparison to the survival observed following transfusion of each population into non-immunized (PBS) recipients evaluated in parallel. Normalized survival for HOD CD47 KO not calculated for time points at which PBS controls reach 0% survival. **(E)** Bar graph representation of normalized survival of transfused HOD CD47 WT, HOD CD47 HET and HOD CD47 KO RBCs 4 hours following transfusion. ns = not significant. *p<0.05, **p < 0.01, ***p < 0.001.

With HOD donor RBCs that express distinct levels of the CD47, we next examined the consequence of transfusing these unique RBC donor populations into recipients immunized with anti-HEL antibodies. To accomplish this, HOD CD47 WT, CD47 HET or CD47 KO RBCs were labeled with the fluorescent dye, DiI, as outlined previously, to facilitate detection post-transfusion (**Figures 2A, B**). HOD negative RBCs were labeled with a fluorescently distinct dye, DiO, as a control (**Figures 2A, B**). Consistent with previous findings, transfusion of HOD CD47 WT RBCs into recipients with anti-HEL antibodies failed to impact RBC clearance, regardless of the specific anti-HEL antibody used or even when recipients received a combination of the anti-HEL antibodies (**Figures 2C-E**). When HOD CD47 HET RBCs were transfused into non-immunized recipients, despite expressing only half the level of CD47, no appreciable difference in RBC survival was detected (**Figures 2C-E**). Similar results were observed when these same HOD CD47 HET RBCs were transfused into recipients passively immunized with only one anti-HEL monoclonal antibody (**Figures 2C-E**). In contrast, when HOD CD47 HET RBCs were transfused into recipients immunized with a combination of anti-HEL antibodies, accelerated clearance could be detected (**Figures 2C-E**). These results suggest that CD47 may regulate RBC sensitivity to antibody-mediated removal.

To determine whether the complete absence of CD47 impacts the outcome of antibody-engagement on HOD RBC clearance, we next employed the exact same strategy of labeling HOD CD47 KO RBCs, followed by transfusion into non-immunized or anti-HEL immunized recipients. In contrast to the HOD CD47 WT or HOD CD47 HET RBCs, which failed to demonstrate noticeable differences in survival post-transfusion in the absence of anti-HEL antibodies, HOD CD47 KO RBCs exhibited reduced survival following transfusion into non-immunized recipients (**Figures 2C-E**). In contrast to the outcome of HOD CD47 HET RBC transfusion, this removal was accelerated following transfusion into recipients immunized with only a single monoclonal anti-HEL antibody (**Figures 2C-E**). The rate of clearance was even more pronounced following HOD CD47 KO transfusion into recipients immunized with both anti-HEL antibodies, where this difference was more apparent when normalizing to clearance rates observed in the absence of anti-HEL antibodies (**Figures 2C-E**). Taken together, these results suggest that CD47 may not only regulate RBC survival in general but may also influence the consequence of antibody engagement on RBC clearance following an incompatible transfusion.

**Figure 3:**
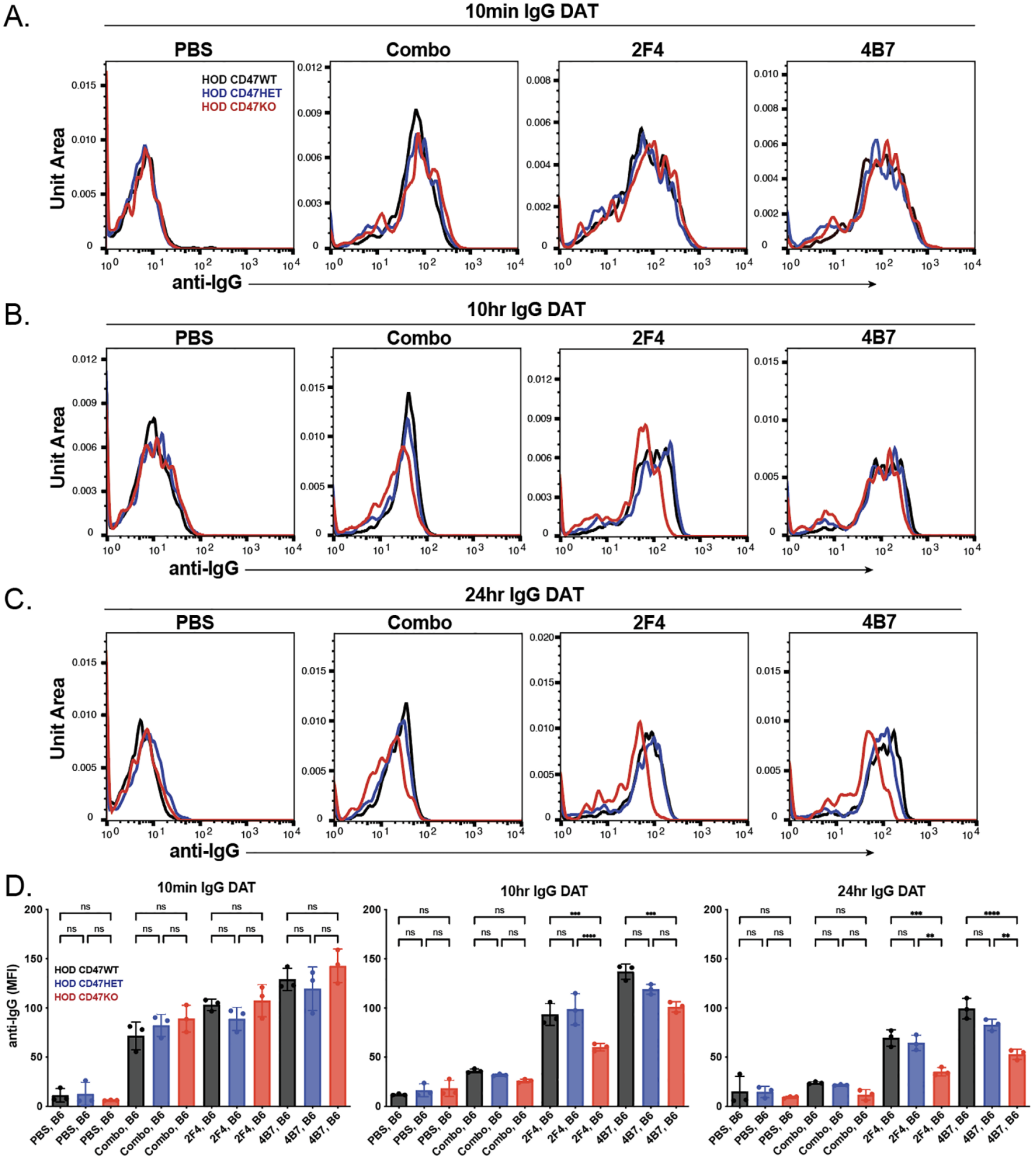
The levels of anti-HEL antibodies decrease over time following transfusion of HOD CD47 WT, heterozygous and knockout RBCs into immunized recipients. Detection of antibody levels on the surface of HOD CD47 WT, HOD CD47 heterozygous (HET) or HOD CD47 knockout (KO) RBCs following transfusion into non-immunized (PBS) or recipients immunized with a single (2F4 or 4B7) or combination (combo - 2F4 and 4B7) of anti-HEL antibodies shown as histograms at 10 minutes **(A)**, 10 hours **(B)**, and 24 hours **(C)** following transfusion. **(D)** Quantification of histogram MFI values of antibody bound on HOD CD47 RBCs WT, HOD CD47 HET and HOD CD47 KO RBCs at 10 minutes, 10 hours, and 24 hours post-transfusion. ns = not significant. **p<0.01, ***p < 0.001, ****p < 0.0001.

While analysis of anti-HEL antibody engagement of each HOD RBC donor population demonstrated similar binding activity *in vitro*, to determine whether differences in HOD RBC CD47 WT, HOD CD47 HET and HOD CD47 KO clearance following transfusion into each immunized recipient may reflect differences in antibody binding *in vivo*, we next examined antibody engagement at various time points following transfusion. Consistent with the levels of antibody engagement observed following incubation with each monoclonal anti-HEL antibody *in vitro*, no significant differences in antibody binding could be detected 10 minutes following transfusion into each immunized recipient (**Figures 3A, D**). In contrast to the enhanced antibody levels observed following incubation of a combination of monoclonal antibodies compared to each monoclonal anti-HEL antibody alone *in vitro*, decreased antibody binding was observed following transfusion into recipients with a combination as opposed to a single monoclonal anti-HEL antibody (**Figures 3A, D**).

To assess if there was a sustained variation in detectable antibody levels on each population over time, we analyzed antibody levels on each RBC population at later time points. Similar to the observations 10 minutes after transfusion, each HOD donor RBC population transfused into recipients immunized with a single antibody showed higher detectable antibody levels compared to similar HOD donor RBCs transfused into recipients previously immunized with a combination of anti-HEL antibodies (**Figures 3A-D**). Interestingly, though the antibody levels on HOD CD47 WT, HOD CD47 HET, and HOD CD47 KO RBCs were initially comparable post-transfusion, a more pronounced decrease in antibody levels was noted on HOD CD47 KO RBCs in recipients previously immunized with a single anti-HEL antibody (**Figures 3A-D**). This difference in bound antibody levels persisted on each RBC population when examined 24 hours post-transfusion (**Figures 3A-D**).

**Figure 4:**
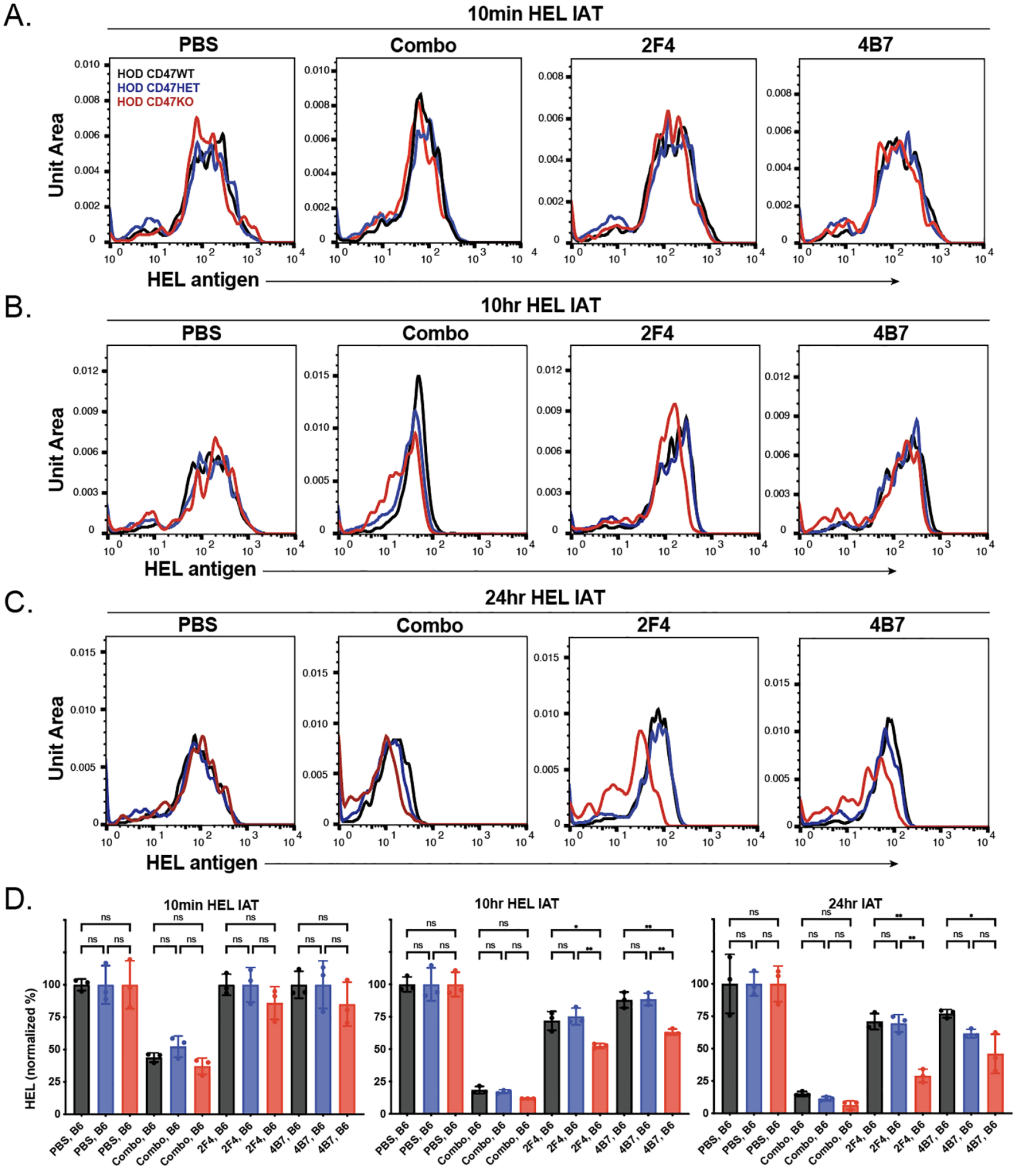
Anti-HEL antibodies reduce HOD antigen levels on HOD CD47 WT, heterozygous and knockout RBCs. Detection of HOD antigen levels on the surface of HOD CD47 WT, HOD CD47 heterozygous (HET) or HOD CD47 knockout (KO) RBCs following transfusion into non-immunized (PBS) or recipients immunized with a single (2F4 or 4B7) or combination (combo - 2F4 and 4B7) of anti-HEL antibodies shown as histograms at 10 minutes **(A)**, 10 hours **(B)**, and 24 hours **(C)** following transfusion. **(D)** Quantificaiton of histogram MFI values of HOD antigen on HOD CD47 RBCs WT, HOD CD47 HET and HOD CD47 KO RBCs at 10 minutes, 10 hours, and 24 hours post-transfusion. ns = not significant. *p<0.05, **p<0.01.

Previous data indicated that the reduction in antibody on the surface of antigen-positive RBCs following transfusion can be due to antibody-mediated alterations in the target antigen itself (77–79, 87, 101, 102). To explore this possibility, we analyzed the levels of the HOD antigen in recipients at various time points post-transfusion. We observed no significant change in the HEL antigen levels shortly after transfusion across different types of HOD RBCs (HOD CD47 WT, HOD CD47 HET, and HOD CD47 KO) in recipients who were immunized with a single anti-HEL antibody (**Figures 4A, D**). However, when each RBC HOD donor population was transfused into recipients who had been immunized with multiple anti-HEL antibodies, significant reductions in the levels of detectable anti-HEL antibodies were noted (**Figures 4A, D**). Similar to antibody levels, antigen levels continued to decline over time following transfusion into recipients with anti-HEL antibodies, with the rate and magnitude of decreases in antigen detection being more pronounced in the face of a combination of anti-HEL antibodies than individual anti-HEL antibodies alone (**Figures 4A-D**). These results align with prior results demonstrating that a combination of anti-HEL antibodies is more efficient at inducing reductions in the levels of detectable antigen (77, 78, 87).

**Figure 5:**
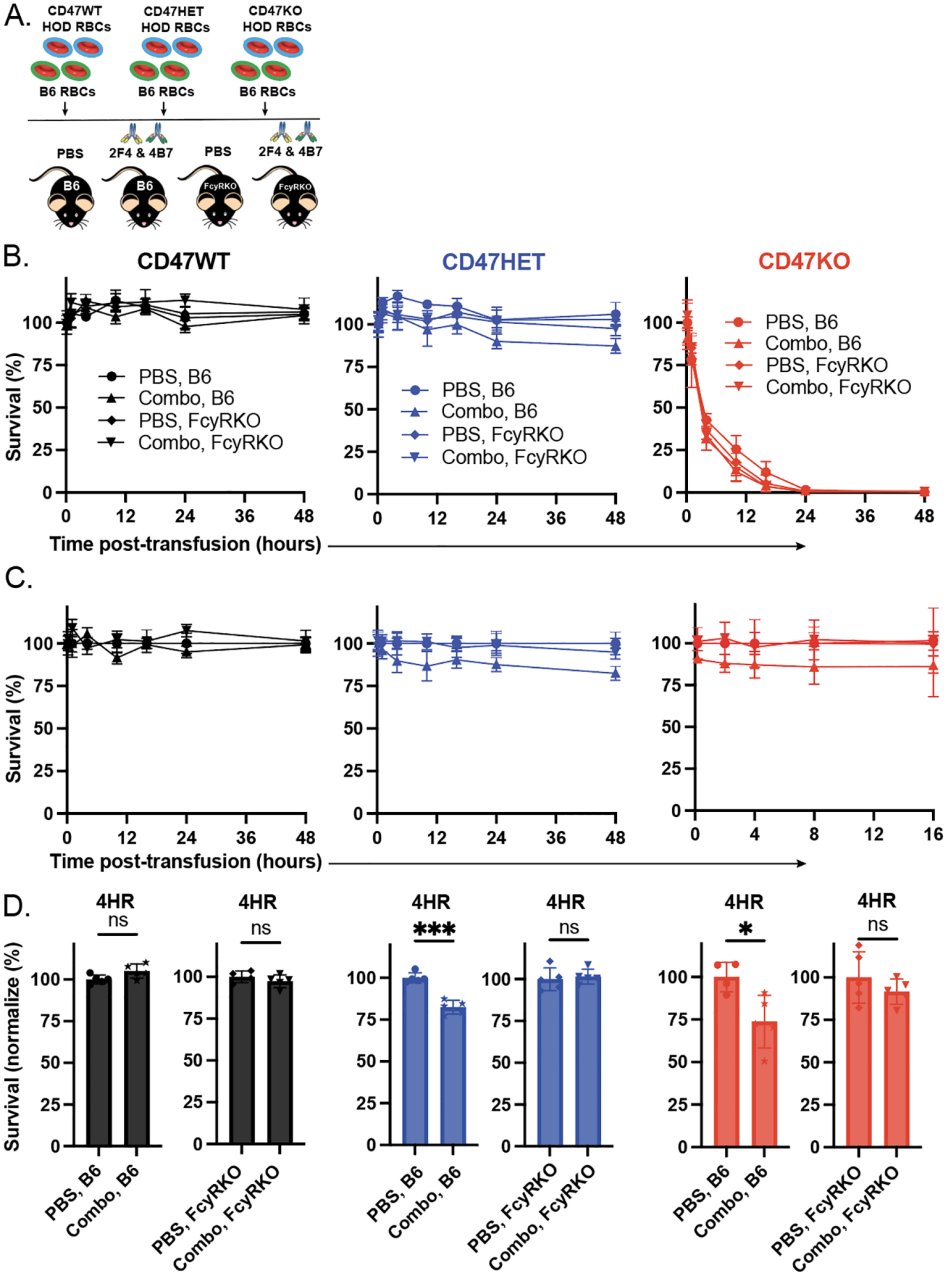
Enhanced antibody-mediated clearance of HOD CD47 heterozygous and knockout RBCs requires Fcγ receptors. **(A)** Depiction of the experimental design using HOD CD47 WT, HOD CD47 heterozygous (HET) or HOD CD47 knockout (KO) RBCs transfused into WT or Fc**γ** receptor (FcγR) KO recipients that were either non-immunized (PBS) or immunized with a single (2F4 or 4B7) or combination (combo - 2F4 and 4B7) of anti-HEL antibodies. **(B)** Survival of transfused HOD CD47 WT, HOD CD47 HET and HOD CD47 KO RBCs over time in comparison to B6 RBCs in the same recipient following transfusion into either WT or FcγR KO recipients that were either non-immunized (PBS) or immunized with a single (2F4 or 4B7) or combination (combo - 2F4 and 4B7) of anti-HEL antibodies. **(C)** Normalized survival of transfused HOD CD47 WT, HOD CD47 HET and HOD CD47 KO RBCs over time in comparison to B6 RBCs in the same recipient following transfusion into either WT or FcγR KO recipients that were either non-immunized (PBS) or immunized with a single (2F4 or 4B7) or combination (combo - 2F4 and 4B7) of anti-HEL antibodies. Normalized survival for HOD CD47 KO not calculated for time points at which PBS controls reach 0% survival. **(D)** Bar graph representation of normalized survival of transfused HOD CD47 WT, HOD CD47 HET and HOD CD47 KO RBCs in comparison to B6 RBCs in the same recipient following transfusion into either WT or FcγR KO recipients that were either non-immunized (PBS) or immunized with a single (2F4 or 4B7) or combination (combo - 2F4 and 4B7) of anti-HEL antibodies 4 hours following transfusion. ns = not significant. *p<0.05, ***p < 0.001.

Prior studies demonstrated that elimination of RBCs can occur through FcγR-dependent and -independent mechanisms (77, 78, 87). To define whether the augmented HOD RBC clearance observed following transfusion of HOD CD47 HET and HOD CD47 KO RBCs into immunized recipients reflects an FcγR-dependent or -independent process, we examined the clearance of HOD RBCs following transfusion into non-immunized or immunized WT or FcγR KO recipients. As the most pronounced differences in antibody-mediated clearance of HOD CD47 HET and HOD CD47 KO RBCs occurred in recipients immunized with both monoclonal antibodies, a combination of anti-HEL antibodies were used to passively immunize each recipient. In contrast to the enhanced clearance of HOD CD47 HET RBCs observed following transfusion into immunized WT recipients, similar increases in antibody-mediated clearance were not observed following transfusion into immunized FcγR KO when evaluated in parallel (**Figures 5A-D**). Similarly, antibody-mediated acceleration of HOD RBC CD47 KO removal likewise failed to occur in FcγR KOs when compared to WT recipients (**Figures 5A-D**).

**Figure 6:**
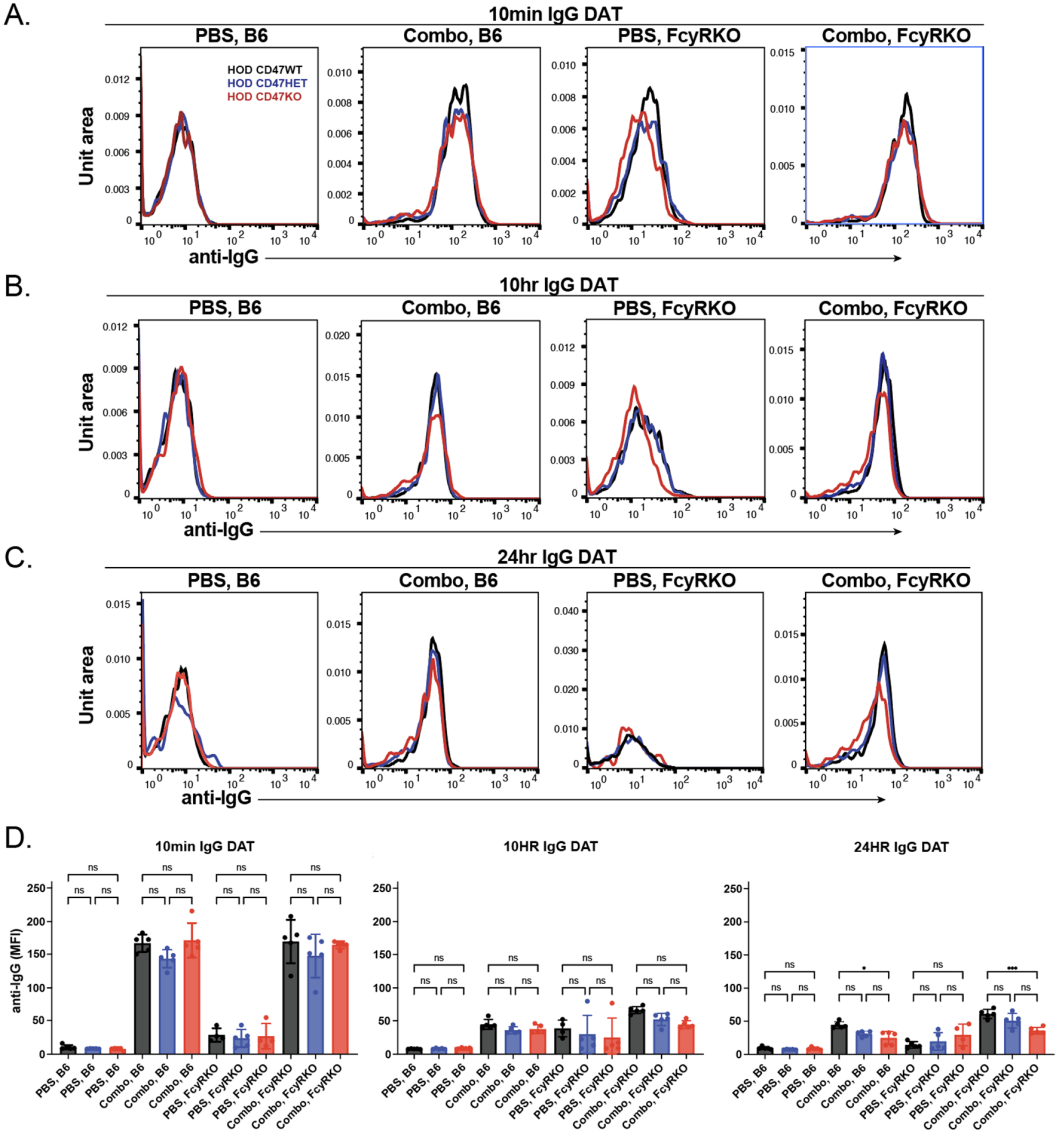
The levels of bound anti-HEL antibodies decrease over time following transfusion of HOD CD47 WT, heterozygous and knockout RBCs into WT and Fcγ receptor immunized recipients. Detection of antibody levels on the surface of HOD CD47 WT, HOD CD47 heterozygous (HET) or HOD CD47 knockout (KO) RBCs following transfusion into WT or Fcγ receptor (FcγR) KO recipients that were either non-immunized (PBS) or immunized with a combination (combo - 2F4 and 4B7) of anti-HEL antibodies shown as histograms at 10 minutes **(A)**, 10 hours **(B)**, and 24 hours **(C)** following transfusion. **(D)** Quantification of histogram mean fluorescence intensity (MFI) values of antibody bound on HOD CD47 WT, HOD CD47 HET and HOD CD47 KO RBCs following transfusion into either WT or FcγR KO recipients that were either non-immunized (PBS) or immunized with a combination (combo - 2F4 and 4B7) of anti-HEL antibodies, 10 minutes, 10 hours, and 24 hours post-transfusion. ns = not significant. *p<0.05, ***p < 0.001.

To determine whether any differences in antibody binding could be observed following transfusion of HOD CD47 WT, HOD CD47 HET, and HOD CD47 KO RBCs into anti-HEL immunized WT or FcγR KOs recipients, we next examined antibody levels on the RBC surface. Similar to results obtained following transfusion of each HOD RBC population into anti-HEL immunized WT recipients, significant reductions in the levels of detectable antibody were observed following transfusion into anti-HEL immunized FcγR KOs recipients when evaluated in parallel (**Figures 6A-D**). Importantly, no difference in antibody levels were observed regardless of whether HOD CD47 WT, HOD CD47 HET, or HOD CD47 KO RBCs were transfused into immunized recipients(**Figures 6A-D**), suggesting that alterations in clearance did not reflect alterations in antibody levels over the same time period.

**Figure 7:**
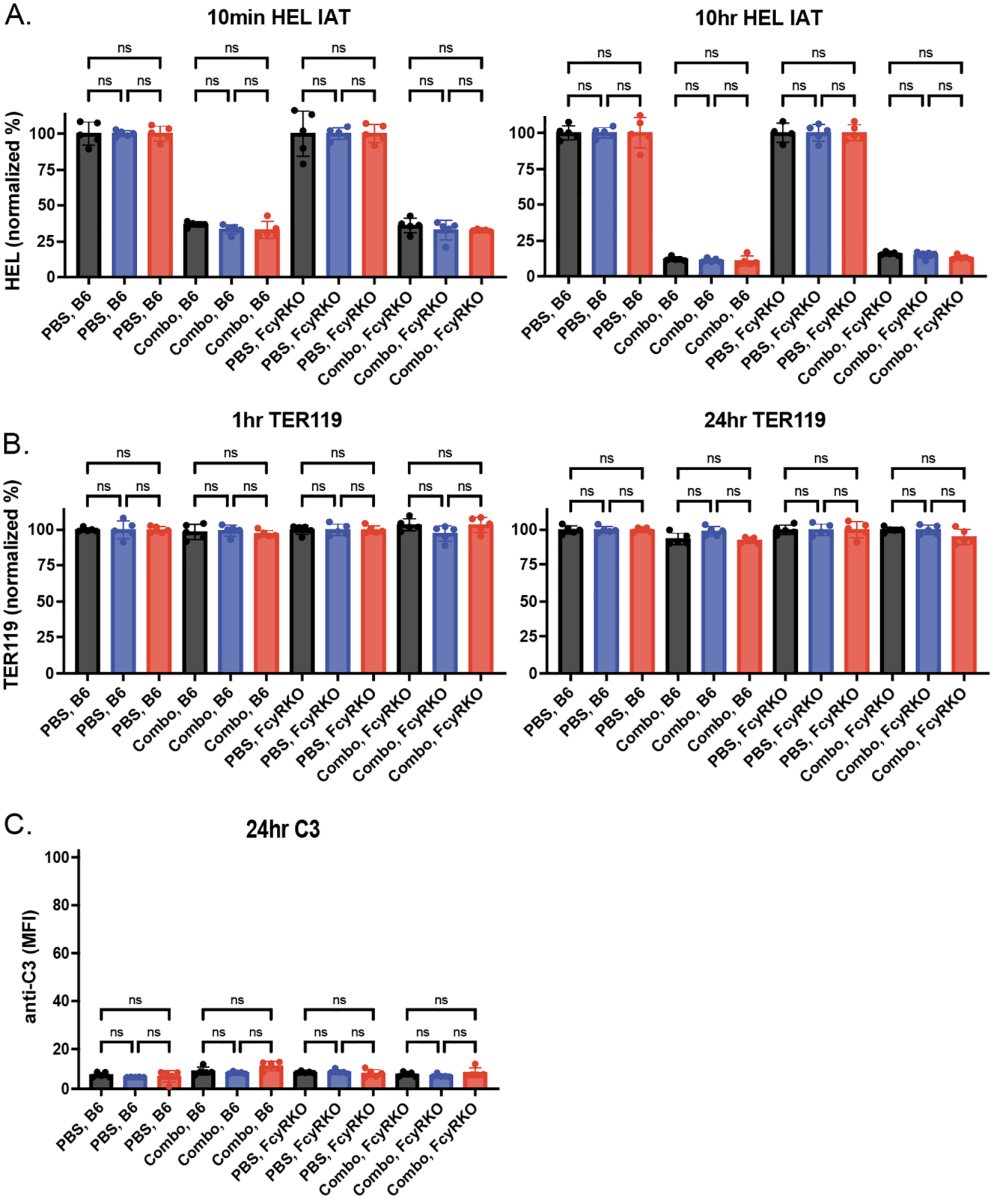
Anti-HEL antibodies reduce levels of HOD without changing Ter119 levels or inducing complement deposition. **(A)** HOD antigen levels on the surface of HOD CD47 WT, HOD CD47 heterozygous (HET) or HOD CD47 knockout (KO) RBCs following transfusion into WT or Fc**γ** receptor (FcγR) KO recipients that were either non-immunized (PBS) or immunized with a combination (combo - 2F4 and 4B7) of anti-HEL antibodies shown as histograms at 10 minutes and 10 hours post-transfusion. **(B)** Levels of Ter119 on the surface of HOD CD47 WT, HOD CD47 HET or HOD CD47 KO RBCs following transfusion into WT or FcγR KO recipients that were either non-immunized (PBS) or immunized with a combination (combo - 2F4 and 4B7) of anti-HEL antibodies shown as histograms at 1 hour and 24 hours post-transfusion. **(C)** Measurement of C3 deposition on the surface of HOD CD47 WT, HOD CD47 HET or HOD CD47 KO RBCs following transfusion into WT or FcγR KO recipients that were either non-immunized (PBS) or immunized with a combination (combo - 2F4 and 4B7) of anti-HEL antibodies shown as histograms 24 hours post-transfusion. ns = not significant.

We next sought to determine whether changes in antibody levels on each HOD RBC population following transfusion into WT or FcγR KOs recipients may be accompanied by alterations in the HOD antigen. To this end, we examined HOD antigen levels on transfused RBCs post-transfusion. Similar to the reductions in antibody levels observed following transfusion into each anti-HEL immunized recipients, the levels of the HOD antigen correspondingly decreased on each HOD RBC population following transfusion into anti-HEL immunized WT or FcγR KO recipients (**Figure 7A**). To determine whether the loss of antigen in each setting was specific to HOD, we next examined whether similar changes in antigen levels occurred to other RBC surface structures. To test this, we examined Ter119, a common marker used to distinguish RBCs from other cellular populations. While levels of detectable antibody and antigen levels decreased over time in immunized recipients, similar changes in Ter119 were not observed (**Figure 7B**. Consistent with the role of FcγR in the antibody-mediated clearance what was observed following HOD RBC het and HOD RBC KO transfusion, very little detectable complement deposition could be observed (**Figure 7C**).

## Discussion

Our findings reveal that CD47 plays a role in controlling RBC removal and antibody modulation following antibody engagement. These data offer new insights into how key features of RBC might influence their vulnerability to removal following antibody engagement. The ability of CD47 to regulate antibody-mediated removal appears to be dosage dependent, with CD47 levels half that of WT rendering HOD RBCs sensitive to antibody mediated removal while failing to significantly impact RBC survival following transfusion into non-immunized recipients. In contrast, complete loss of CD47 not only impacted HOD RBC survival in the absence of anti-HEL antibodies but appears to further sensitize HOD RBCs to additional antibody-mediated removal through a FcγR-dependent process. Taken together, these data suggest that CD47 may be a key feature of RBCs that regulates the consequences of antibody-mediated removal following an incompatible transfusion.

Due to the unpredictable and often severe outcomes of incompatible blood transfusions, deliberately transfusing patients for the express purpose of defining the outcome of incompatible RBC transfusions is not ethical. However, as some patient populations are particularly prone to developing alloantibodies that can make it very difficult to find fully compatible RBCs (1, 15), defining the key regulators of RBC removal following an incompatible transfusion is key for patients for whom fully compatible blood may not be readily available in a timely manner. To this end, there have been a variety of studies that have leveraged preclinical models to define the impact and outcome of incompatible RBC transfusions (74–76). Early studies utilizing RBCs that express the HEL antigen (membrane bound HEL or mHEL) first identified the ability of antibody engagement to modulate the target antigen in the absence of detectable RBC removal using this approach (77, 78). These studies were followed by similar findings in other model systems, such as the HOD system used in the present study and in model systems that employ distinct target antigens, such as KEL and GPA (74–76, 79–87). In each of these systems, antibody engagement possessed the ability to specifically modulate the target antigen, although the rates of antigen modulation and the overall outcome on RBC survival can differ. These results suggest that the ability of antibodies to modulate a target antigen following engagement may reflect a common outcome that can occur following an incompatible transfusion, though the rate and degree of antigen modulation can vary. It is important to note that similar patterns have been observed in patients treated with antibody-based therapies that target RBC antigens, such as CD38 and RhD (101, 102), suggesting that these observations aren’t limited to preclinical models.

The discovery that antibodies can induce antigen modulation suggests that leveraging this outcome could enhance alterations in the target antigen over RBC elimination in settings where fully compatible RBCs are not available. However, for such possibilities to be realized, the mechanisms responsible for regulating antigen loss over RBC clearance must first be established. Among the natural regulators of RBC removal, CD47 is one of the most well-studied. Initially identified on RBCs as an inhibitor of RBC clearance (95), the interaction between CD47 and SIRPα has been extensively shown to inhibit cellular removal. Cancer cells often exploit CD47 to decrease phagocytic removal, prompting the development of therapies targeting CD47 to improve immune detection and elimination (98). However, the specific influence of CD47 in the context of antibody-driven RBC removal after mismatched transfusions remains relatively unexplored. The differential sensitivity of HOD CD47 HET RBCs to antibody-mediated removal demonstrates that even decreased levels of CD47 can influence the outcome of antibody engagement on RBC survival. Further studies will be needed to determine whether increased levels of CD47 would further protect RBCs otherwise sensitive to antibody-mediated removal from FcγR-mediated clearance. Various iPSC-derived RBC strategies and other forms of RBC modification could possibly be leveraged to enhance the ability of CD47 to inhibit RBC clearance following an incompatible transfusion (103, 104). Manipulation of the CD47-SIRPα axis in the recipient may also represent an alternative avenue to enhance RBC survival following an incompatible transfusion, although much more research needs to be done to determine the feasibility of these possibilities.

The capacity of CD47 to modulate the elimination of RBCs following antibody binding is intriguing and likely reflects to the interplay between the antibody-induced activation of phagocytes via FcγRs and the inhibitory effect of CD47-SIRPα on phagocytosis. Data consistent with this general concept was published decades ago (105, 106), although the molecular mechanisms responsible for regulating phagocytosis remained unknown. Our data suggest that the balance between the inhibitory presence of CD47 and pro- phagocytic effects mediated by FcγR pathways is a key regulator. The ability of antibody engagement to induce antigen modulation likely reflects an evolutionarily conserved pathway whereby antibody bound antigenic material attached to the RBC surface is delivered to sentinel immune cells (107–109). In this setting, the accumulation of immune complexes on the surface of RBCs may provide a mechanism of antigen delivery from distant sites, thereby facilitating a rapid response to blood-borne infection (107–109). The development of alloantibodies following pregnancy or transfusion, followed by re-exposure of RBCs with the target antigen in the setting of clinical practice may mimic this same process. However, in the setting of transfusion, the bolus of RBCs received may quickly overwhelm the ability of antigen modulation to remove antibody-antigen complexes, in contrast to directly causing RBC removal.

While most patients with a positive direct antiglobulin test fail to exhibit significant signs of hemolysis (110), patients with various forms of autoimmune hemolytic anemia clearly possess antibodies capable of causing RBC removal. As a result, a balance between the potency of the antibody to facilitate RBC clearance versus antigen modulation may dictate the ultimate outcome of antibody-antigen interactions on the RBC surface. As CD47 is known to regulate the rate of RBC clearance, it may reflect one factor that regulates this balance between RBC removal and modulation of antigen on the RBC surface following antibody engagement. Interestingly, CD47 can modulate complement, as well as FcγR-dependent cell removal (97). While we have focused on the HOD model in our current study, future studies examining the role of CD47 in other established incompatible RBC transfusion models will provide intriguing insight in the full regulatory potential of CD47 in this setting. In addition to preclinical models of RBC alloimmunization, the recent development of novel models of autoimmune hemolytic anemia will certainly aid in understanding these relationships (111–113).

Although differences in reticulocyte number could theoretically contribute to the rate and magnitude of HOD RBC clearance observed in this study, differences in reticulocytes percentages between HOD CD47 WT versus HOD CD47 KO among the overall donor units were subtle. Consistent with this, there was a lack of a detectable difference in total HEL levels in the peripheral blood among units tested from HOD CD47 WT, HOD CD47 HET and HOD CD47 KO donors. Indeed, only a 1% difference in the total reticulocytes was observed in the total RBC population between HOD CD47 WT versus HOD CD47 KO units. As nearly 25% of the transfused RBCs cleared following antibody engagement of HOD CD47 HET or HOD CD47 KO RBCs, if all the reticulocytes cleared following antibody engagement, it is unlikely that a possible preferential reticulocyte clearance could account for all the antibody-mediated clearance observed following transfusion of HOD CD47 HET or CD47 KO RBCs into immunized recipients. However, it is possible that reticulocytes impact this effect through other, unknown mechanisms and that differences in reticulocyte content may contribute to subtle differences in overall RBC clearance.

While our current work is focused on the effects of CD47 levels on RBC clearance and antigen-modulation following an incompatible transfusion, levels of CD47 may also play an important role in regulating alloimmune responses following transfusion, including antibody-mediated immunosuppression. Changes in the duration of RBC circulation and antigen levels, both of which are modulated by CD47 in the current model, have been suggested to be key factors in the induction of an alloimmune response (114). Additionally, differences in RBC unit composition (%reticulocyte) in HOD CD47 KO when compared to HOD CD47 WT, but not in HOD CD47 HET donors, could also play a role in alloimmunization. Reticulocytes in general displayed a higher level of HOD antigen expression. Whether this difference impacts alloimmunization of each unit in the presence or absence of anti-HEL antibodies remains to be tested. As recent studies have suggested a higher immunogenic potential for reticulocyte-rich RBC units (51), this remains a distinct possibility and should be investigated in future studies.

As with any study, the current study is not without limitations. First and foremost, this is a preclinical model and may or may not reflect similar outcomes following incompatible RBC transfusion in patients. However, as noted previously, given limitations in the ability to deliberately expose patients to incompatible RBCs due to the inability to predict outcomes, similarly defining the impact of antibody engagement following incompatible transfusion clinically remains difficult to routinely study. In cases where patients have received antibody-based treatments that target RBCs, antigen loss has occurred (101, 102). However, whether CD47 regulates this process remains unknown and will require further study. The potential role of CD47 on RBC removal and antigen loss following antibody engagement of target antigens employed in other models of incompatible RBC transfusion biology will also need to be examined. As RBCs have been noted to experience differences in CD47 upon storage (115), this was the primary focus of the present study. Similarly, while our current study design strove to isolate variable levels of CD47 on transfused RBCs, whether global differences in CD47 levels of recipients may also play a role in RBC clearance or alloimmunization following transfusion remains to be explored. While such studies will require the development of similar genetic deletion models of CD47 on distinct RBC model antigen backgrounds, such studies will be important to define the extent to which CD47 regulates distinct forms of antibody-mediated RBC removal. Despite these limitations, the present results suggest that, at least in the HOD model, CD47 may govern the consequences of antibody engagement with implications in key factors that regulate the outcome of incompatible RBC transfusion.

## Data Availability

The raw data supporting the conclusions of this article will be made available by the authors, without undue reservation.
