# Supplementary material for: CD47 regulates antigen modulation and red blood cell clearance following an incompatible transfusion
Source: Front Immunol. 2025 Apr 4;16:1548548. doi: 10.3389/fimmu.2025.1548548 (PMC12006802; doi:10.3389/fimmu.2025.1548548)
Supplement: Supplementary file 1 [file Image1.pdf]

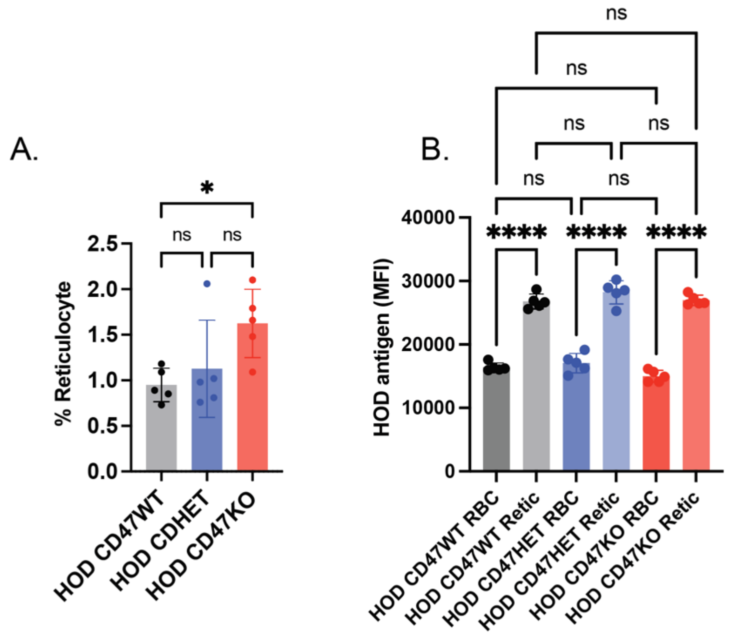

Supplemental Figure 1. Analysis of the peripheral blood composition in HOD CD47 WT, HET or KO donors. (A) Reticulocytes percentage within the Ter119+ RBC population were identified in peripheral blood collected from HOD CD47 WT, HOD CD47 HET, or HOD CD47 KO donors. (B) Levels of the HOD antigen on mature RBCs or reticulocytes from HOD CD47 WT, HOD CD47 HET, or HOD CD47 KO donors as indicated. ns = not significant, $*p<.05$, $****p<.0001$
